# Supplementary material for: De Novo Transcriptome Meta-Assembly of the Mixotrophic Freshwater Microalga Euglena gracilis
Source: Genes (Basel). 2021 May 29;12(6):842. doi: 10.3390/genes12060842 (PMC8227486; doi:10.3390/genes12060842)
Supplement: Supplementary file 1 [file genes-12-00842-s001.zip › Cordoba-2021-Euglena-Supplementary-Materials-v2/Cordoba-2021-Euglena-HTML-S3-Krona-cpETC.html]

Javascript must be enabled to view this page.

magnitude
magnitudeUnassigned

Euglena\_ETC\_cpt

33

32

30
4

19
1

2
9

4

1
4

1

1

1

1

2

2

2

3

3

3

3

3

3

9

8

8

8

8

8

1

1

1

1

1

1

1

1

1

1

1

1

1

1

1

1

1

6

1

1

1

1

1

1

4

4

4

4

4

4

1

1

1

1

1

1

1

1

1

2

2

2

2

1

1

1

1

1

1

1

1

1

1

1
