# Supplementary material for: De Novo Transcriptome Meta-Assembly of the Mixotrophic Freshwater Microalga Euglena gracilis
Source: Genes (Basel). 2021 May 29;12(6):842. doi: 10.3390/genes12060842 (PMC8227486; doi:10.3390/genes12060842)
Supplement: Supplementary file 1 [file genes-12-00842-s001.zip › Cordoba-2021-Euglena-Supplementary-Materials-v2/Cordoba-2021-Euglena-HTML-S1-Krona-global.html]

Javascript must be enabled to view this page.

magnitude
magnitudeUnassigned

Euglena\_transcripts\_all

49919

36069

937
13850

11196
4054

18
375

158

158
41

60

60

60

60

57

57

57

57

199

199

199

199

199

199

468
29

139

139

139

139

300
12

187

187

187

101

101

101
1

100

530
25

327

327

327

327

178

178

178

178

178

1364

1364
285

809

809

809

80
270

94

94

94

94

94

96

96

96

96

96

96

265

265

265

265

926
115

42

42

42

42

198

198
48

45

45

45

55

55

50

50

50

19
148

109

109
18

39

39

39

39

52

8
52

19

19

25

25

20

20

20

20

20

20

262
92

13

13

13

13

36

36

36

36

92

92

92

92

92

29

29

29

29

90

90

90

90

90

90

63

63

63

63

63

8

8

8

947
136

9

9

9

9

9

35

35

35

35

35

427

73
427

27
219

135

135

135

135

135

135

135

135

135

135

135

135

135

135

135

135

135

135

135

135

135

135

135

135

135

57

57

57

57

57

57

57

57

135

135

135

135

135

135

115

115

115

115

115

22
225

13
161

15

15

15

13

13

13

13

13

13

13

25

25

25

25

25

25

11
95

54

54

54

54

54

54

30

30

30

30

30

30

30

30

30

42
6

24

24

10

10

10

10

10

10

10

10

14

14

14
1

3

3

3

10

10

10

10

2
12

7

7

7

7

7

7

7

1

1

1

1

1

1

2

2

2

2

2

2

2

2

119
977

47
432

78

78

78

78

78

199

36
199

62

62

62

62

13
101

59

59

29

29

108

108

108

108

108

426
78

148

148

148

148

148

200

200

45
200

88

88

88

88

88

88

88

88

88

88

88

88

88

88

67

67

67

67

67

847
38

10
201

101

101

101

90

90

90

19

14

14

14

1
14

11

11

11

11

2

2

2

2

5

5

5

5

5

5

5

37

37

37

37

37

37

37

417

417

417
71

123

123

223

223

135

135
1

20

20

20

20

20

20

20

20

114

114

114

114

114

114

279

13

13

13

13

13

2
266

130

130

130

130

134

134

134

134

134

164
5

7
104

32

32

32

32

13

2
13

7

7

4

4

52

1
52

31
3

20

8

20

20

55

55

55

55

55

55

27
2

17

13
1

6

6

1
4

2
1

1

1

1

1

2

2

1

1

1

1

6

3

2

1

1

1

1

1

1

2
3

1

1

1

2

2

2

1

1

1

1

1

1

1

1

1

1

1

1

1

1

1

1

1

1

1

8

6

1
6

2

2

1

1

1

1

3

3

3

3

3

2

1

1

1

1

1

1

1

451
1690

1

1

1

1

1

452
29

1

1

1

1

1

1

1

1

1

1

1

1

1

12

12
1

8

5

5
4

1

1

3

3

3

3

3

3
1

2

2

2

65

1

1

1

1

1

1

11
60

1

1

1

1

1

1

1

1

4

1
4

1

1

1

1

1

8

1

1

1

1

1

1

1

1

1

1

1

1

2
1

1

1

1

1

1

1

2

2

1

1

1

1

2

1

1

1

1

1

1

1

7

7

7
2

1

1

1

2

23
3

8
3

1

1

2

2

2

2

3

3

2

2

1

1

2

2

1

1

1

7
1

4

4

2

1

1

1

2

2

2

2

2

1

1

1

1

1

1

1

1

1

1

1

3

3

2

2

2

2

1

1

1

212

81
212

3
30

3

3

3

3

3

3

3

3

2

2

2

3

3
2

1

3
13

1

1

1

1

1

1

3
8

1

1

1

2

3

3

2

1

6
29

1

1

1

1

1

1

1

3

3

2

2

1

1

8

2

2

2

6

1

1

1

4

4

4

1

1

1

1

1

1

2

2

2

6
1

1

1

1

2

2

2

2

1

1

1

3

3

3

1
3

2

2

1
69

60
8

2

1

1

1

1

1

7

7

7

7

7

7

2

2

2

1

1
13

2

2

2

9

5

5

4

4

1

1

23
1

6

1

5

5

2

2

2

4

4

1

1

1

3
9

2

2
4

2

8
1

3

3

1

1

2

2

4

4

2
4

1

1

91
8

1

1

1

1

1

1

1

1

1

1

1

1

1

1

1

1

1

1

1
28

1

1

1

1

1

1

25

10

10
5

1

1

1

1

1

1

1

1

4

1

1

1

3

3

6

1

1

1

1

1

2

2

2

1

1

1

1

1

1

1

1

1

1

1

1

1

1

2

2

2

2

52
1

4
45

2

2

1

1

1

1

1

1
11

1
3

1

1

1

1

7
2

1

2
1

1

1

1

1

1

1

3

3

1

1

1

1

25

6
1

3

3

2

19
6

1

1

1

4

5

1

1

1

1

1
6

1

1

1

1

2

2

1

1

1

1

2

2

2

2

2

40

3

3

3

3

3

3

3

3

26

3

3

3

3

3

23
3

11

10

10

8

2

2

1

1

1

1

9

9

3
9

6

4

4

4

4

4

4

6

6

6

6

6

6

1

1

1

1

1

1

1

1

1

1

2

2

1
2

1

1

1

1

1

1

1

1

1

1

1

1

1

1

1

1

1

1

1

1

1

40

11

2

2

2

2

2

9

9

2

2

2

2

7
1

1

1

1

1

1

1

1

1

1

1

1

1

1
2

1

1

15

15
1

4

4

4

4

1
10

1

1

1

1

2
7

4

4

1

1

1

1

1

1

1

1

1

1

1

1

1

13
1

4
1

3

3

2

2

2

1

1

3

3

3

3

3

3

5

4

3

3

3

3

1

1

1

1

1

1

110
576

3

1

1

1

1

1

2

2

2

2

124
22

9

1

1

1

1

8

1

1

1

1

1

1
2

1

1

2

2

1

1

1

1

13
1

2

2

2

1

1

1

3

1

1

1

2

2

2

6

6

1
6

4

1

1

7

1

1

1

3

3

3

3

3

3

1
26

17
2

4

4

4

2

1

1

1

1

1

2

1

1

1

2
4

1

1

2

2

2

1

1

1

8
2

1

1

1

4
1

3

1

1

1

1

1

5
37

1

1

15

13

1

1

2
12

1

1

1

3

3

5

5

1

1

1

1

1

1

1

5

1

1

1

1

1

1

1

1

2

1

1

1

1

1

2

2

1

1

1

5

3
5

2

3
1

2
1

1

1

3

1

1

2

1

1

1

1

1

5

2

1

1

1

1

3

1

1

1

1

1

1

1

1

1

31
147

8

7

4

2

2

2

2

2

1

1

1

1

1

2

2
1

1

1

1

1

1

1

12

10

2

2

2

2

2

6

6

2

1

1

1

1

1

21

9

9

1

1

1

7

7

6

1

12

8
12

1

1

1

1

1

1

1

1

1

1

1

1

1

3

3

1

1

1

7

1

1

1

1

1
6

1

1

1

2

2

2

2
1

1

1

1

1

1

1

6

6

2
6

2

2

1

1

15

5
15

3

2

1

1

1

1

2

2
1

1

1

1

1

2

2

2

1

1

1

1

1

1

4
17

1

1

1

4

2
4

2

2

2

2

1
2

1

4

1

1

1

1

1

1

1

1

1

1

1

1

2

2
1

1

1

6

6
1

1

1

1

2
4

2

16
1

2

2

2

2

2

2

1

1

1

6
2

1

1

1

2
3

1

1

4

4

1

2

1

129
2

124
9

2
23

14
6

8

8

7
2

1

1

1

2

2

2

1

1

1

1

1

1

11

6

5

5

5

1

5

5

5

5

2

2

2

8
2

5
1

4
1

1

1

1

1

1

1

1

1

1
5

4

4
1

1

1

1

1

1

1

4
66

2

2

2

22

11

11

11

11
1

3

3

3

7

7
3

1

3

3
27

3

3

3

3
15

5

5

5

3

3

3

3

3

1

1

1

6

6
2

1

1

3

3

11

2

2

2

2

9

9

9

9

3

3

1

1

1

1

2

1

1

1

1

62
8

44
6

5

1

1

4

1

1

1

2

5

1
2

1

1

1

2
1

1

1

4

1

1

1

1

1

1

1

2
20

1

1

1

1
2

1

1

2

1

1

1

1

1

1
12

2
1

1

1

9
1

1
7

2

4

1

1

4
3

1

3

1

1

1

1

1
2

1

1

1
3

1

1

1

1

1

1

3

1

1
2

1

1

1

1

1

1

1

1

1

1

1

108

1

1

1

1

1

1

107
1

3

3

3
1

2

2

2

2

28
91

3

3

3

1

1

1

1

1

1

1

1

1

2

2

2

1

1

1

1

1

4

4

4
1

1

1

1

2

2

2

17

2
17

2

2

2

2

5

3

3

3

2

2

2

1

1

1

1

1

1

1

1

3
1

1

1

1

3
2

1

1

1

2
16

5
14

2

2

2

2

1

1

6

3
6

3

16

16
2

14
1

1

1

1

1

1

1

1

1

1

4
1

1

2

2

2

1

1

1

2

2

2

5

5

1

1

4

4

12

12

12

11
5

2
1

1

1

3

3

3

1

1

1

1

1

1

1

5

2

2

2

2

2

2

3

3

3

1

1

1

2

2

2

35

35

12

1

11
3

1
3

1
2

1

3

3

1

1

1

1

1

1

23

23
2

10

10

10

7
9

1

1

1

2

2

2

1

1

1

1

1

1

1

6

6

6

10

10

10

10

9

9

1

1
