# Supplementary material for: De Novo Transcriptome Meta-Assembly of the Mixotrophic Freshwater Microalga Euglena gracilis
Source: Genes (Basel). 2021 May 29;12(6):842. doi: 10.3390/genes12060842 (PMC8227486; doi:10.3390/genes12060842)
Supplement: Supplementary file 1 [file genes-12-00842-s001.zip › Cordoba-2021-Euglena-Supplementary-Materials-v2/Cordoba-2021-Euglena-HTML-S2-Krona-mtETC.html]

Javascript must be enabled to view this page.

magnitude
magnitudeUnassigned

Euglena\_ETC\_nmt

86

49

37

1
31

1

1

1

1

5
1

1

1

1

1

1

1

3

1
2

1

1

1

1

1

1

1

1

2

2

2

2

2

2

2

2

2

2

1

1

1

1

1

1

1

1

1
2

1

1

1

1

1

1

1

1

1

1

1

19

19

1
19

6

6

6

6

12

1
12

8

8

8

8

8

3

3

3

3

3

3

6

5

5
1

2

1

1

1

1

1

1

1

1

1

1

1

1

1

1

1

1

1

1

1

1

1

1

1

1

1

1
