# Supplementary material for: De Novo Transcriptome Meta-Assembly of the Mixotrophic Freshwater Microalga Euglena gracilis
Source: Genes (Basel). 2021 May 29;12(6):842. doi: 10.3390/genes12060842 (PMC8227486; doi:10.3390/genes12060842)
Supplement: Supplementary file 1 [file genes-12-00842-s001.zip › Cordoba-2021-Euglena-Supplementary-Materials-v2/Cordoba-2021-Euglena-Supplemental-Figures-v2.pptx]

## Slide 1
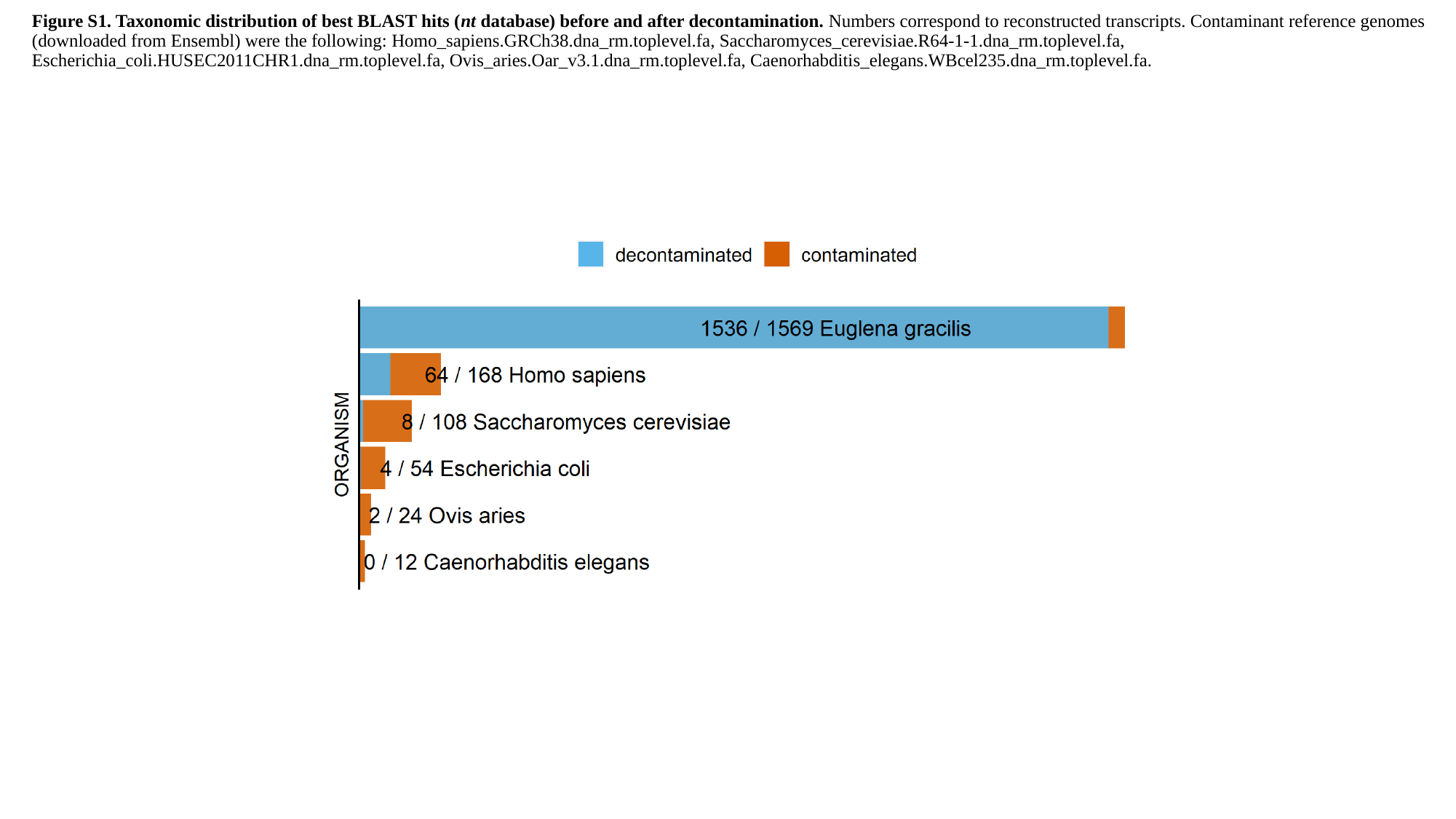

Figure S1. Taxonomic distribution of best BLAST hits (nt database) before and after decontamination. Numbers correspond to reconstructed transcripts. Contaminant reference genomes (downloaded from Ensembl) were the following: Homo_sapiens.GRCh38.dna_rm.toplevel.fa, Saccharomyces_cerevisiae.R64-1-1.dna_rm.toplevel.fa, Escherichia_coli.HUSEC2011CHR1.dna_rm.toplevel.fa, Ovis_aries.Oar_v3.1.dna_rm.toplevel.fa, Caenorhabditis_elegans.WBcel235.dna_rm.toplevel.fa.

## Slide 2
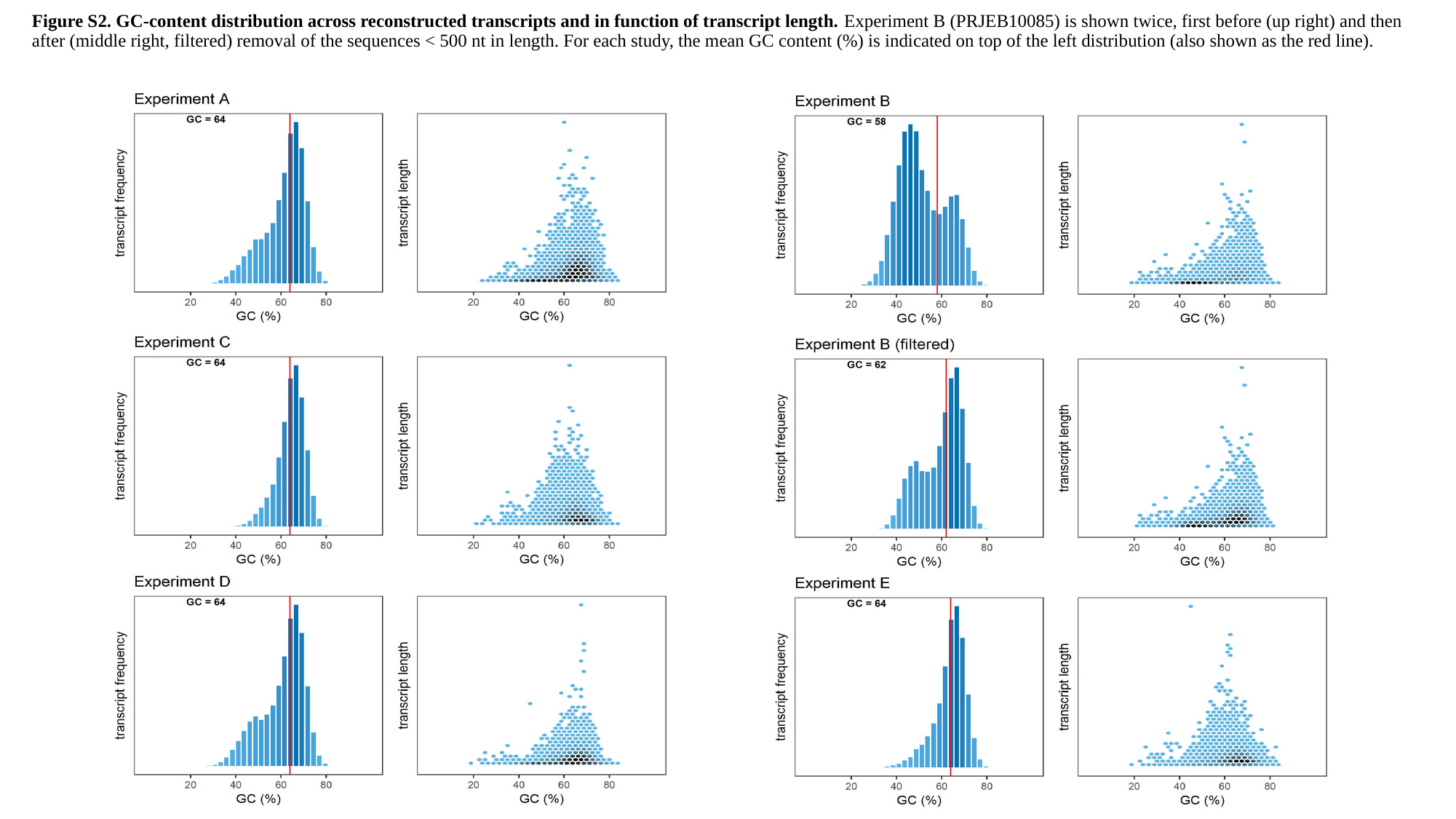

Figure S2. GC-content distribution across reconstructed transcripts and in function of transcript length. Experiment B (PRJEB10085) is shown twice, first before (up right) and then after (middle right, filtered) removal of the sequences < 500 nt in length. For each study, the mean GC content (%) is indicated on top of the left distribution (also shown as the red line).

## Slide 3
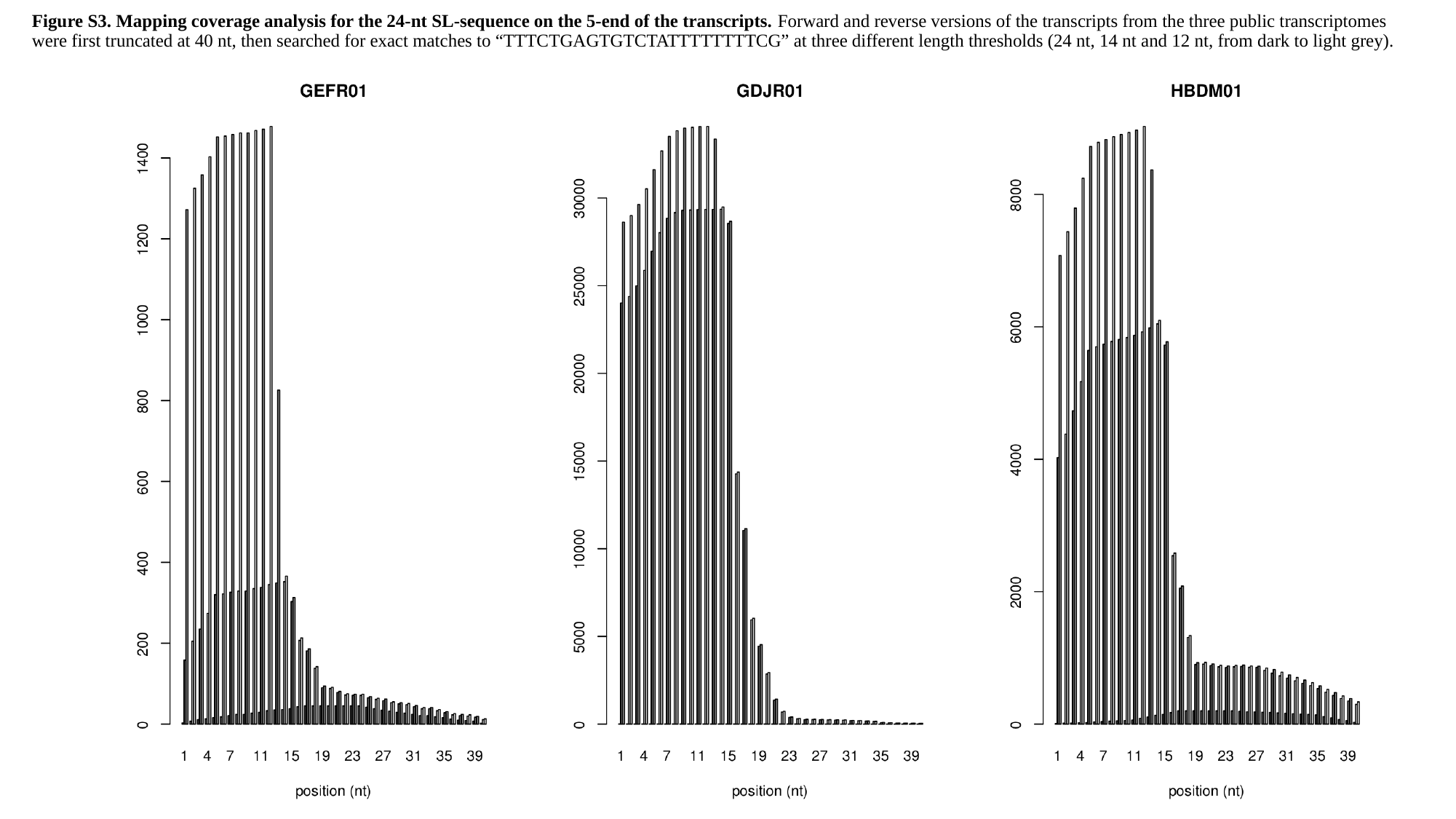

Figure S3. Mapping coverage analysis for the 24-nt SL-sequence on the 5-end of the transcripts. Forward and reverse versions of the transcripts from the three public transcriptomes were first truncated at 40 nt, then searched for exact matches to “TTTCTGAGTGTCTATTTTTTTTCG” at three different length thresholds (24 nt, 14 nt and 12 nt, from dark to light grey).

## Slide 4
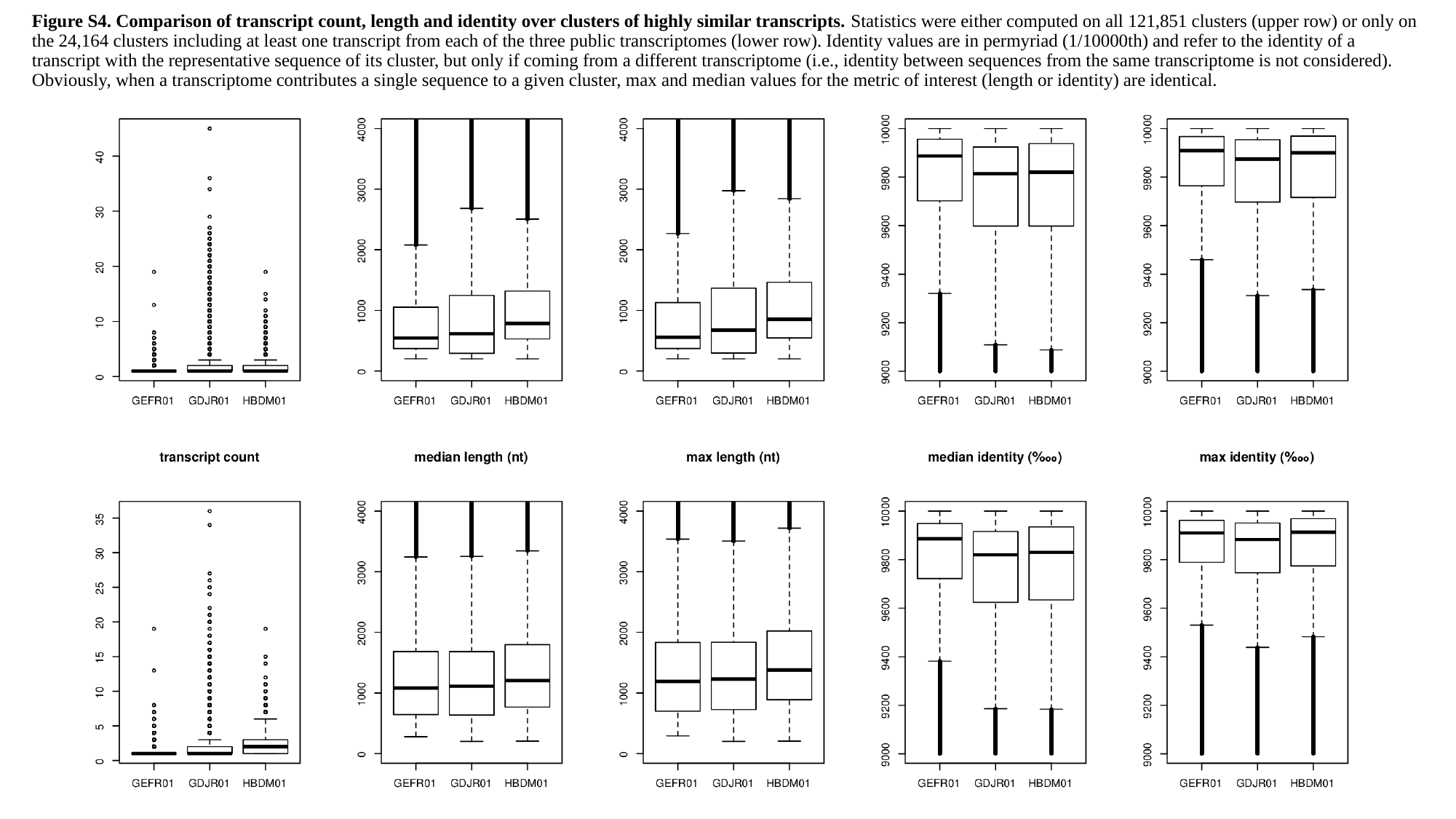

Figure S4. Comparison of transcript count, length and identity over clusters of highly similar transcripts. Statistics were either computed on all 121,851 clusters (upper row) or only on the 24,164 clusters including at least one transcript from each of the three public transcriptomes (lower row). Identity values are in permyriad (1/10000th) and refer to the identity of a transcript with the representative sequence of its cluster, but only if coming from a different transcriptome (i.e., identity between sequences from the same transcriptome is not considered). Obviously, when a transcriptome contributes a single sequence to a given cluster, max and median values for the metric of interest (length or identity) are identical.

## Slide 5
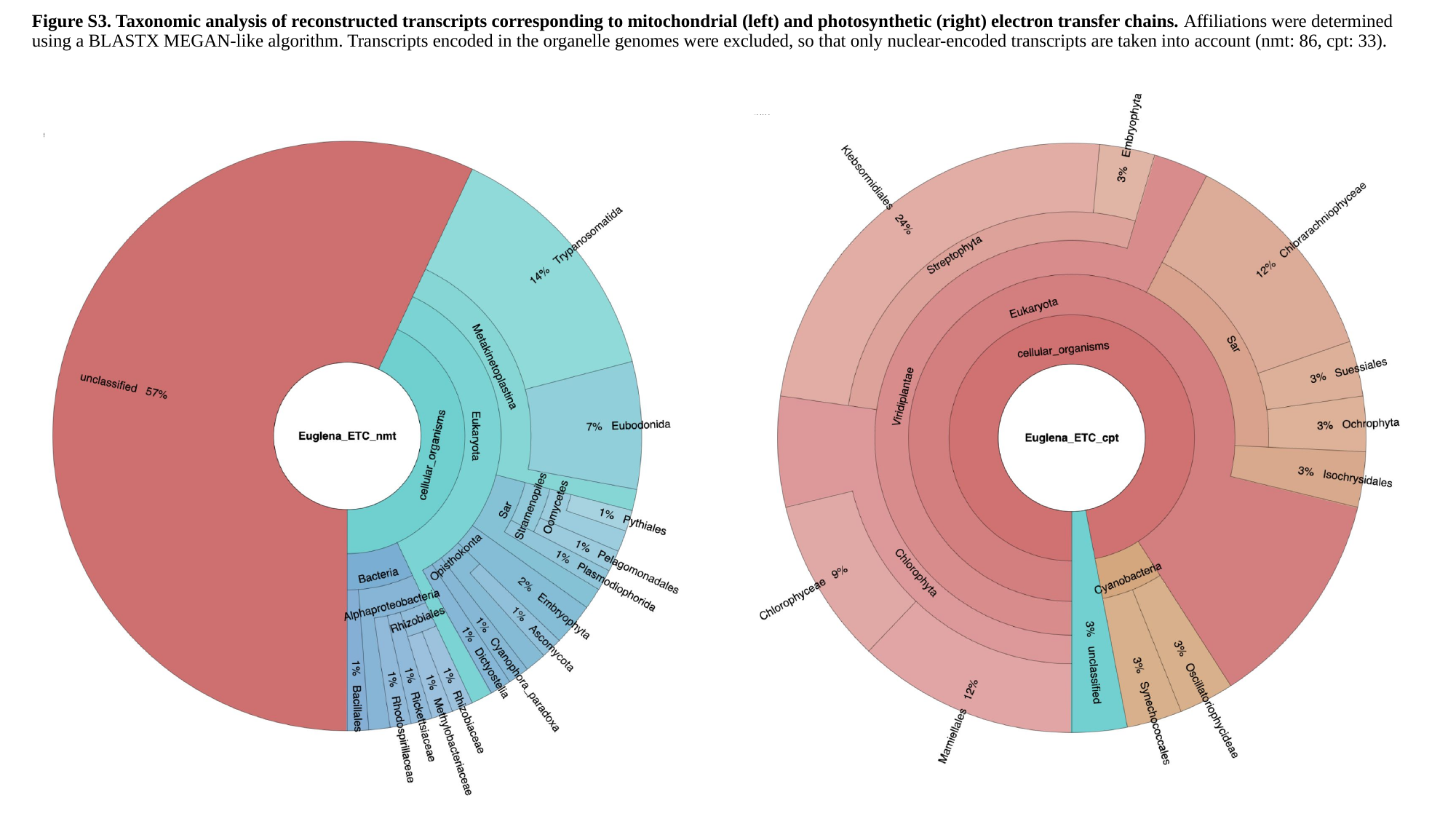

Figure S3. Taxonomic analysis of reconstructed transcripts corresponding to mitochondrial (left) and photosynthetic (right) electron transfer chains. Affiliations were determined using a BLASTX MEGAN-like algorithm. Transcripts encoded in the organelle genomes were excluded, so that only nuclear-encoded transcripts are taken into account (nmt: 86, cpt: 33).

## Slide 6
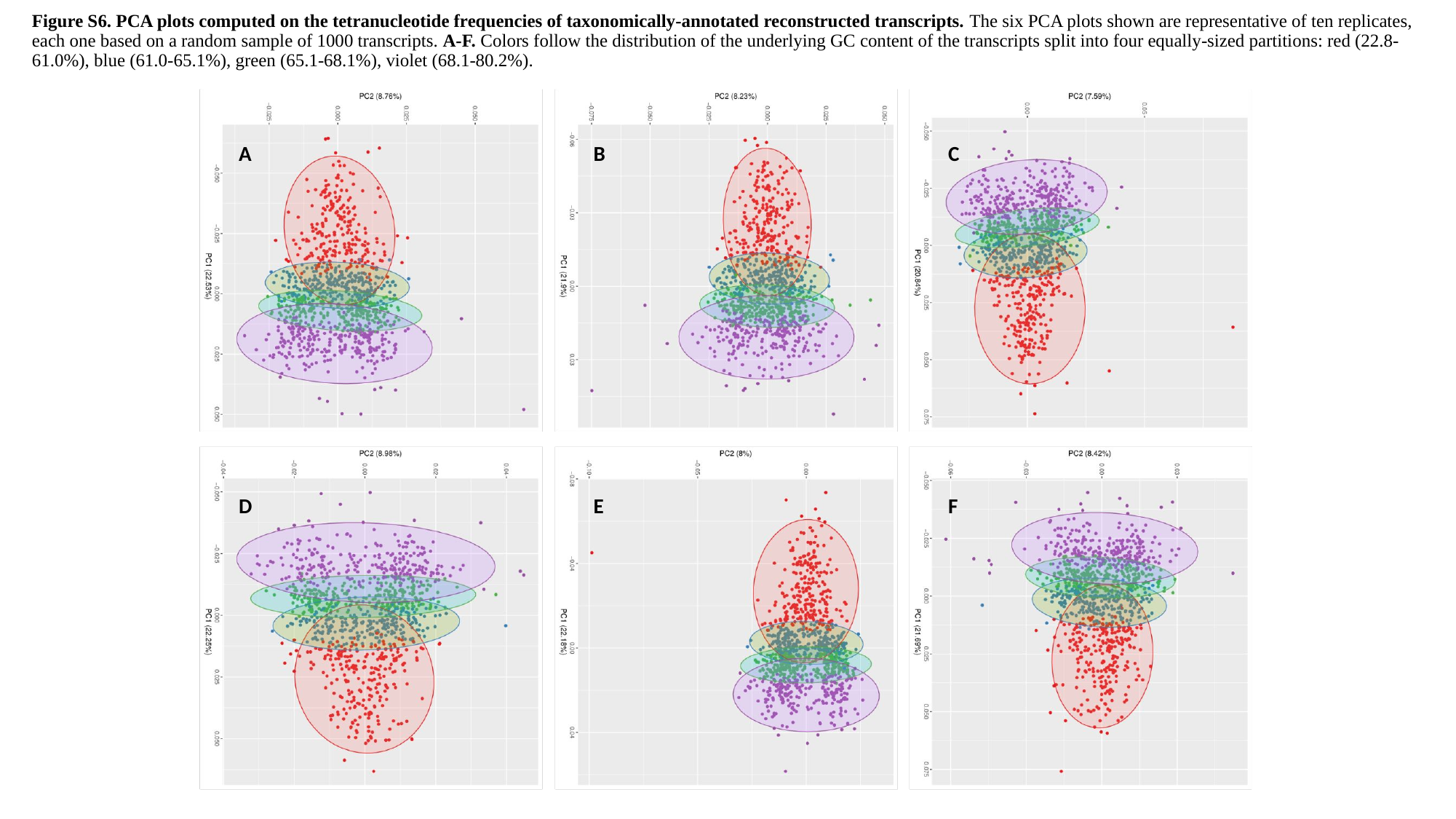

Figure S6. PCA plots computed on the tetranucleotide frequencies of taxonomically-annotated reconstructed transcripts. The six PCA plots shown are representative of ten replicates, each one based on a random sample of 1000 transcripts. A-F. Colors follow the distribution of the underlying GC content of the transcripts split into four equally-sized partitions: red (22.8-61.0%), blue (61.0-65.1%), green (65.1-68.1%), violet (68.1-80.2%).
A
B
C
D
E
F

## Slide 7
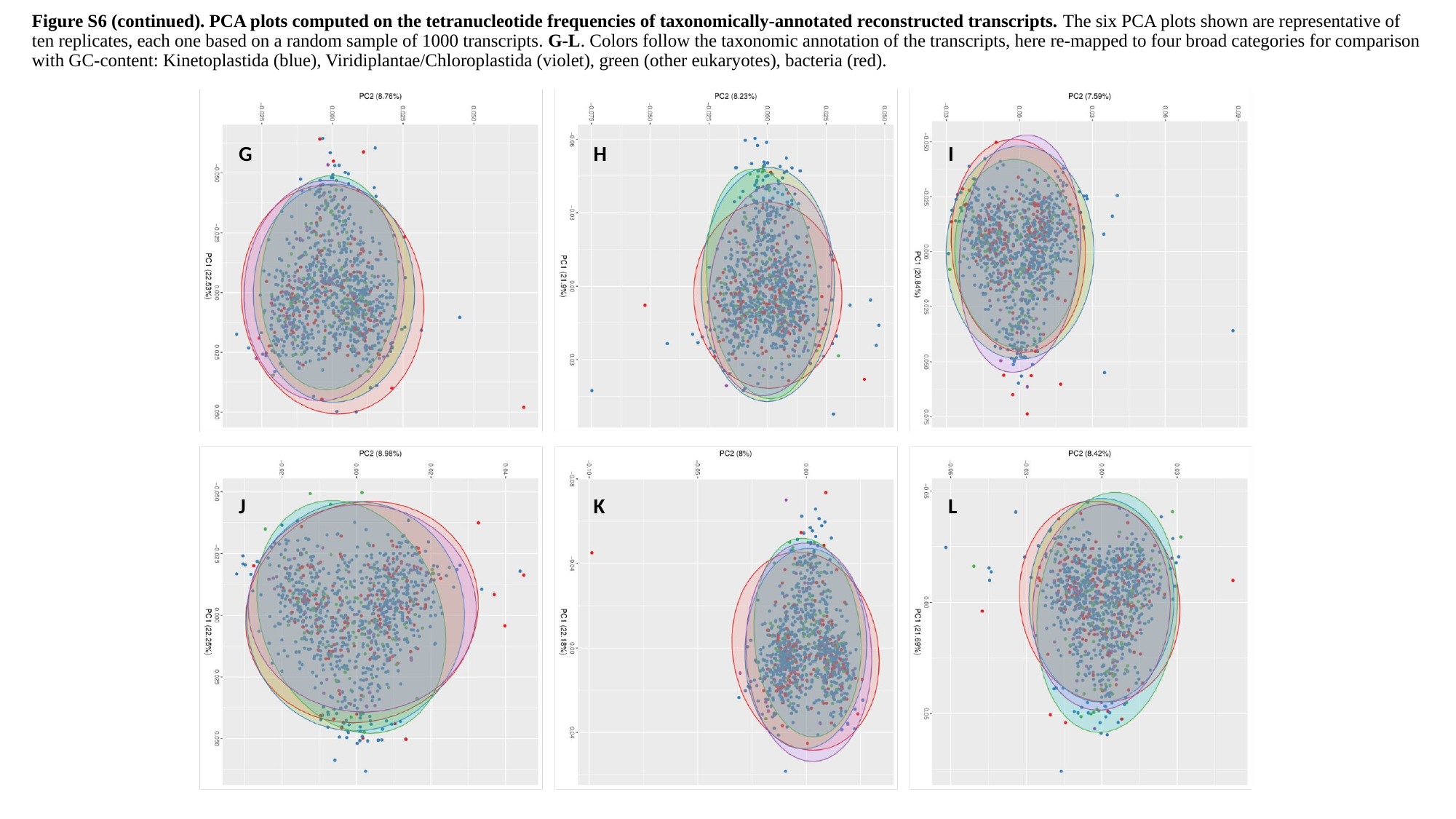

Figure S6 (continued). PCA plots computed on the tetranucleotide frequencies of taxonomically-annotated reconstructed transcripts. The six PCA plots shown are representative of ten replicates, each one based on a random sample of 1000 transcripts. G-L. Colors follow the taxonomic annotation of the transcripts, here re-mapped to four broad categories for comparison with GC-content: Kinetoplastida (blue), Viridiplantae/Chloroplastida (violet), green (other eukaryotes), bacteria (red).
G
H
I
J
K
L

## Slide 8
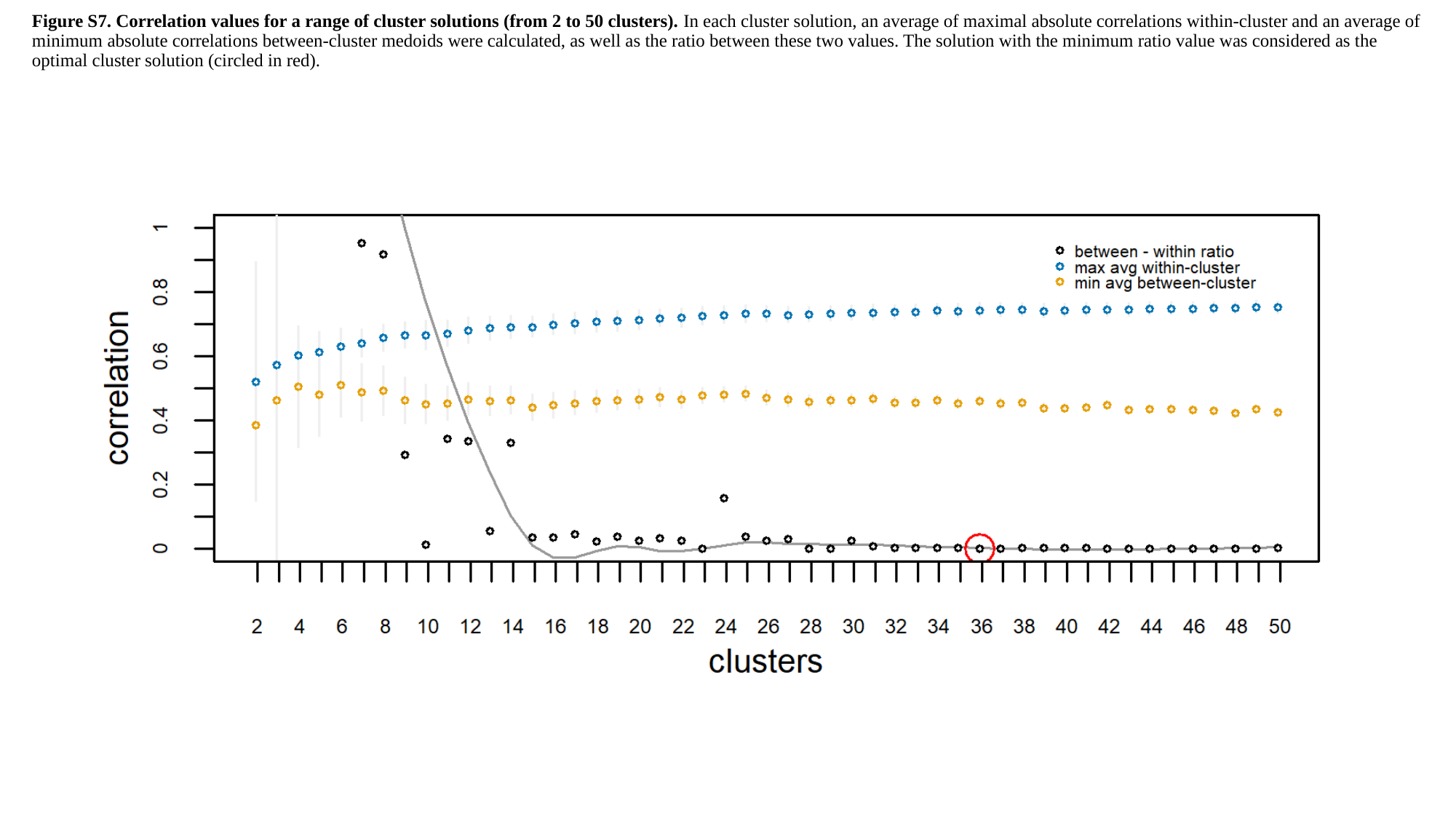

Figure S7. Correlation values for a range of cluster solutions (from 2 to 50 clusters). In each cluster solution, an average of maximal absolute correlations within-cluster and an average of minimum absolute correlations between-cluster medoids were calculated, as well as the ratio between these two values. The solution with the minimum ratio value was considered as the optimal cluster solution (circled in red).

## Slide 9
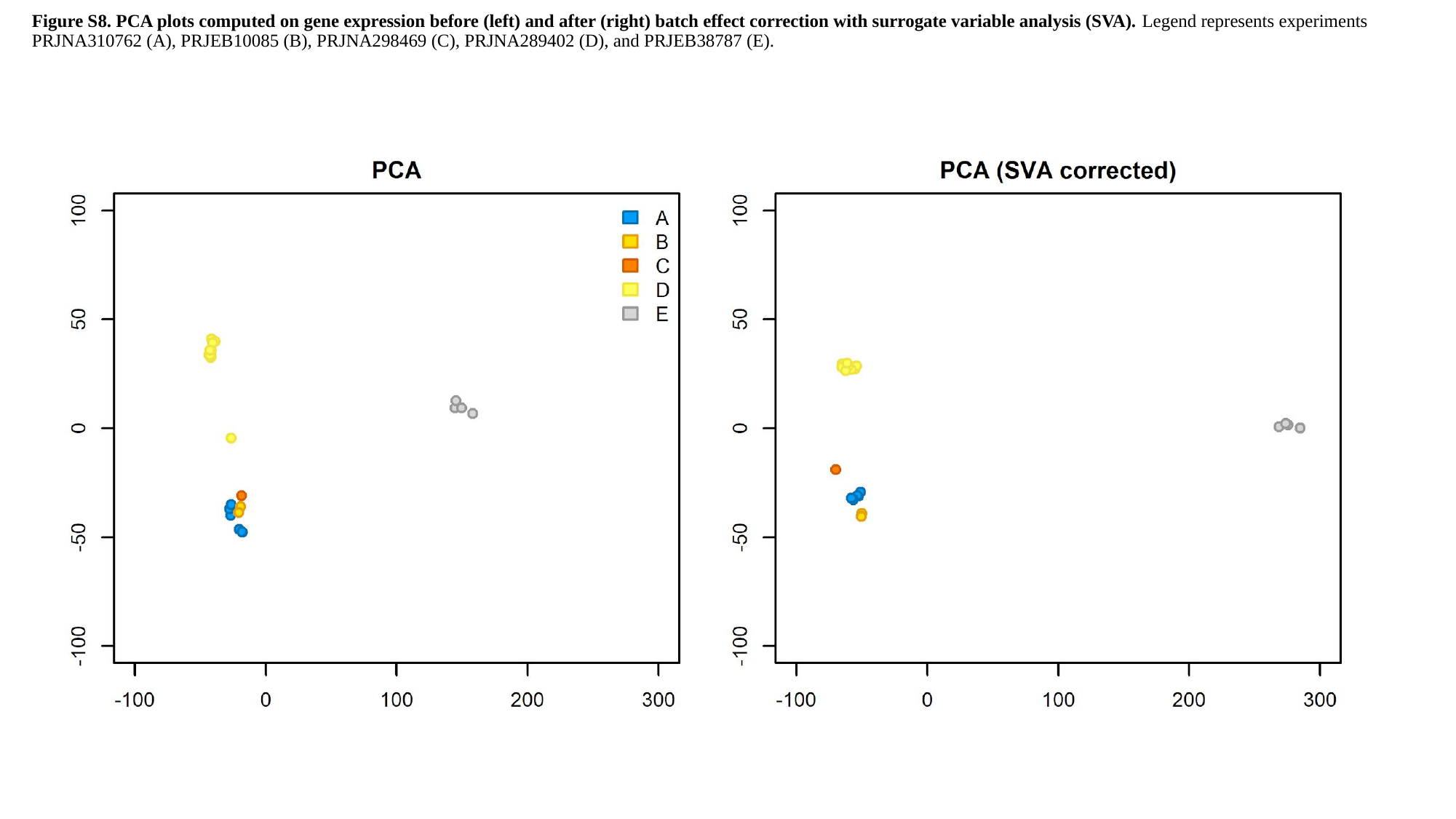

Figure S8. PCA plots computed on gene expression before (left) and after (right) batch effect correction with surrogate variable analysis (SVA). Legend represents experiments PRJNA310762 (A), PRJEB10085 (B), PRJNA298469 (C), PRJNA289402 (D), and PRJEB38787 (E).

## Slide 10
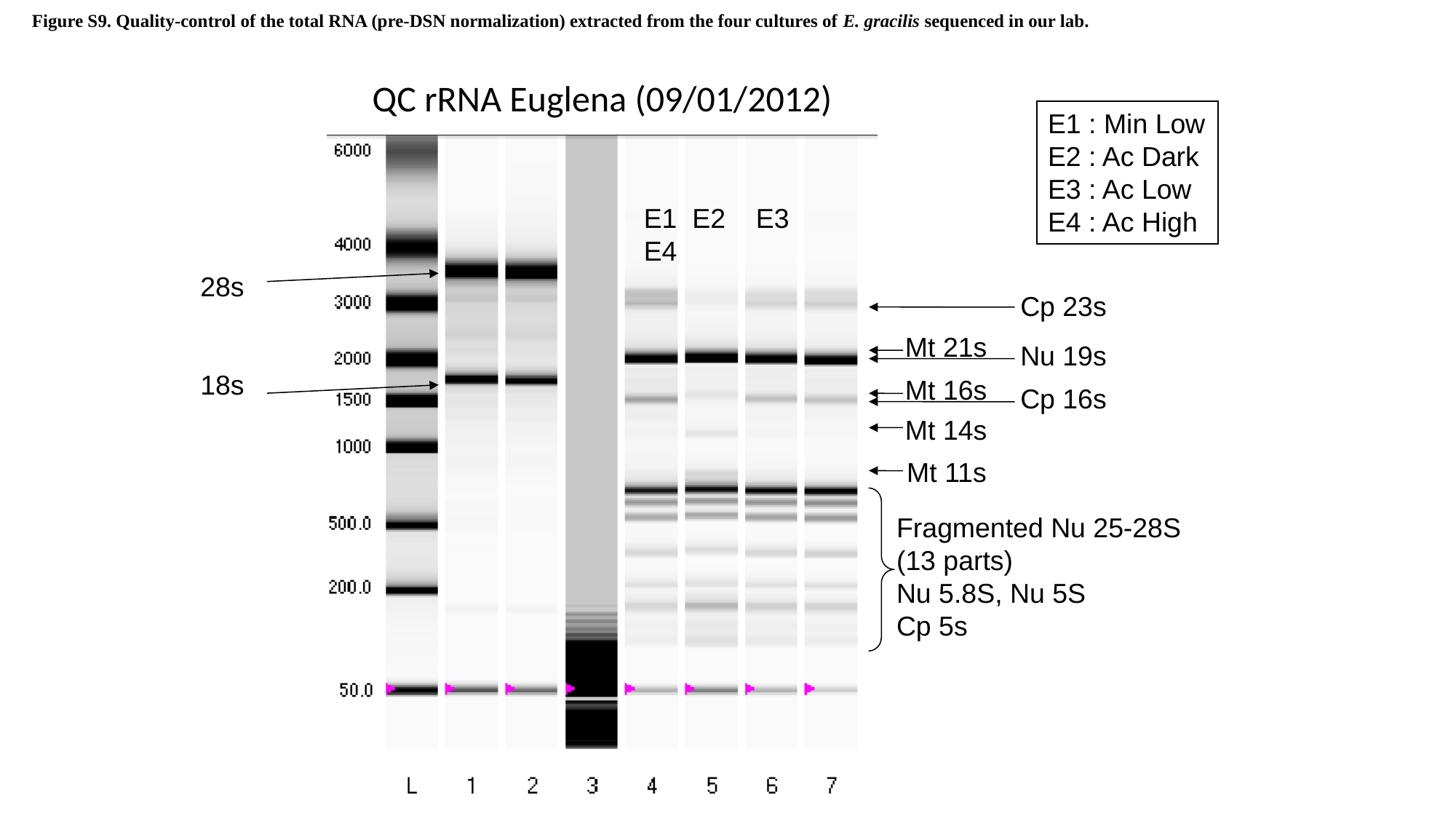

Figure S9. Quality-control of the total RNA (pre-DSN normalization) extracted from the four cultures of E. gracilis sequenced in our lab.
QC rRNA Euglena (09/01/2012)
E1 : Min Low
E2 : Ac Dark
E3 : Ac Low
E4 : Ac High
E1 E2 E3 E4
28s
18s
Cp 23s
Mt 21s
Nu 19s
Mt 16s
Cp 16s
Mt 14s
Mt 11s
Fragmented Nu 25-28S
(13 parts)
Nu 5.8S, Nu 5S
Cp 5s
